# Cancer therapy related complications in the liver, pancreas, and biliary system: an imaging perspective

**DOI:** 10.1007/s13244-015-0436-7

**Published:** 2015-10-06

**Authors:** Danny Ngo, Jemianne Bautista Jia, Christopher S. Green, Anjalie T. Gulati, Chandana Lall

**Affiliations:** Eastern Virginia Medical School, 700 W Olney Road, Norfolk, VA 23507 USA; Department of Radiological Sciences, University of California, Irvine, 101 The City Drive South, Orange, CA 92868 USA

**Keywords:** Cancer therapy, Drug-associated adverse effects, Hepatic toxicity, Pancreatic toxicity, Biliary toxicity

## Abstract

**Abstract:**

Awareness of cancer therapy-induced toxicities is important for all clinicians treating patients with cancer. Cancer therapy has evolved to include classic cytotoxic agents in addition to newer options such as targeted agents and catheter-directed chemoembolisation. Several adverse affects can result from the wide array of treatments including effects on the liver, pancreas, and biliary system that can be visualised on imaging. These complications include sinusoidal obstruction syndrome, fatty liver, pseudocirrhosis, acute hepatitis, pancreatitis, pancreatic atrophy, cholecystitis, biliary sclerosis, and biliary stasis. Many of these toxicities are manageable and reversible with supportive therapies and/or cessation of cancer therapy. The objective of this review is to discuss the imaging findings associated with cancer therapy-induced toxicity of the liver, biliary system, and pancreas.

***Teaching Points*:**

• *Cancer therapy can have adverse effects on the hepatobiliary system and pancreas.*

• *Cancer therapy-induced toxicities can be visualised on imaging.*

• *Knowledge of imaging changes associated with cancer therapy complications can improve treatment.*

## Introduction

Chemotherapy is an essential component in the treatment of cancer. Systemic cancer therapy has evolved from the classic cytotoxic agents to now include newer classes of molecularly targeted therapy. Classic cytotoxic agents target rapidly proliferating cells by interfering with their cell division and growth. Newer therapies target specific cancer molecules involved in regulating cancer cell growth and differentiation. Additionally, the use of chemoembolisation, a minimally invasive procedure used to locally administer chemotherapeutic agents directly to tumours, continues to grow and is predominantly used in the treatment of liver cancers.

While these therapies aim to inhibit cancerous tissue growth, both systemic and localised therapies are known to have adverse effects on normal tissues. With the varying mechanisms of action, it is not surprising that the side effects of different anticancer agents and treatment modalities are diverse, affecting nearly every organ system. Many of the adverse effects of cancer therapy on the liver, pancreas, and biliary system can be detected on imaging (Table 1). These include sinusoidal obstruction syndrome (SOS), fatty liver, pseudocirrhosis, hepatitis, pancreatitis, pancreatic atrophy, cholecystitis, biliary sclerosis, and biliary inflammation. The objective of this article is to review and discuss the imaging findings associated with cancer therapy-related changes in the liver, pancreas, and biliary system and to provide didactic cases.

**Table 1:** Table summarising adverse effects visible on imaging and most commonly associated cancer therapies

Adverse effect	Associated therapy	Laboratory findings	Radiologic findings
Fatty liver	Oxaliplatin, irinotecan, fluorouracil, methotrexate	↑ ALT ↑ AST	US: increased echogenicity and beam attenuation CT: reduced liver attenuation MR: reduced liver signal intensity in out-of-phase imaging
Sinusoidal obstruction syndrome (SOS)	Oxaliplatin, fluorouracil, mercaptopurine, dacarbazine, azathioprine	↑ Bilirubin	US: ascites, gallbladder wall thickening, hepatosplenomegaly CT: ascites, decreased right hepatic vein diameter (<0.45 cm), perioesophageal varices, hepatosplenomegaly, and recanalisation of the umbilical vein
Pseudocirrhosis	Gemcitabine	None	US, CT, MR: segmental volume loss, capsular retraction, fibrosis, enlargement of caudate lobe
Acute hepatitis	Anastrozole, lapatinib	↑ ALT ↑ AST	US: “starry sky” sign CT: Hepatosplenomegaly, thickened gallbladder wall, periportal oedema, decreased liver enhancement
Hepatic abscess	TACE	↑ Alkaline phosphatase Hypoalbunaemia	CT: hypoattenuating lesion with peripheral rim enhancement
Hepatic failure	TACE	Thrombocytopenia ↑ PT, INR ↑ ALT ↑ AST ↑ Bilirubin	US: increased echogenicity, ascites, nodularity, segmental hypertrophy/atrophy CT: surface and parenchymal nodularity, segmental hypertrophy/atrophy
Pancreatitis	L-asparaginase, carboplatin, cisplatin, cytarabine, ifosfamide, paclitaxel, tretinoin, vinorelbine, TACE	↑ Amylase ↑ Lipase	US: peripancreatic fluid collection, hypoechoic lesions CT: areas of low attenuation, diffuse pancreatic oedema
Pancreatic atrophy	Sorafenib, sunitinib	None	CT: reduced pancreatic volume
Acute acalculous cholecystitis	Everolimus, sunitinib, bevacizumab	↑ Alkaline phosphatase ↑ Bilirubin	US: gall bladder wall thickening, gallbladder distension, pericholecystic fluid collection CT: gallbladder distension, fat stranding, hyperaemia, pericholecystic fluid
Biliary inflammation	L-asparaginase, doxorubicin, epirubicin, paclitaxel	None	CT: biliary epithelial thickening and enhancement
Biliary sclerosis	HAIPC w/ floxuridine	↑ Alkaline phosphatase ↑ Bilirubin	CT: thickened/enhanced bile duct wall, bile duct stricture with lumen <3 mm, periductal oedema ERCP: dilatation and stricture of biliary tree
Biliary stasis	Tamoxifen, doxorubicin	↑ Alkaline phosphatase ↑ Gamma-glutamyl transpeptidase	US: biliary dilatation CT: tumefactive sludge
Bile duct injury	TACE	↑ Alkaline phosphatase ↑ Gamma-glutamyl transpeptidase ↑ Bilirubin	CT: main bile duct dilatation, extrabiliary collection of bile MRCP: biliary fluid collection and bile duct leaks

## Effects of systemic therapy

### Liver

#### Fatty liver

Fatty infiltration of hepatic tissue as well as buildup of fat globules in hepatocytes is considered hepatic steatosis while steatohepatitis is a more severe form of fatty liver disease with hepatocyte degeneration. Steatohepatitis is often asymptomatic; however, it can often manifest through elevations of alanine transaminase (ALT) and aspartate transaminase (AST). Fatty changes have been linked to the chemotherapeutic agents oxaliplatin, irinotecan, 5-FU, and methotrexate with initial steatotic appearance occurring from 2 weeks to 2 months after therapy [1, 2].

**Fig. 1:**
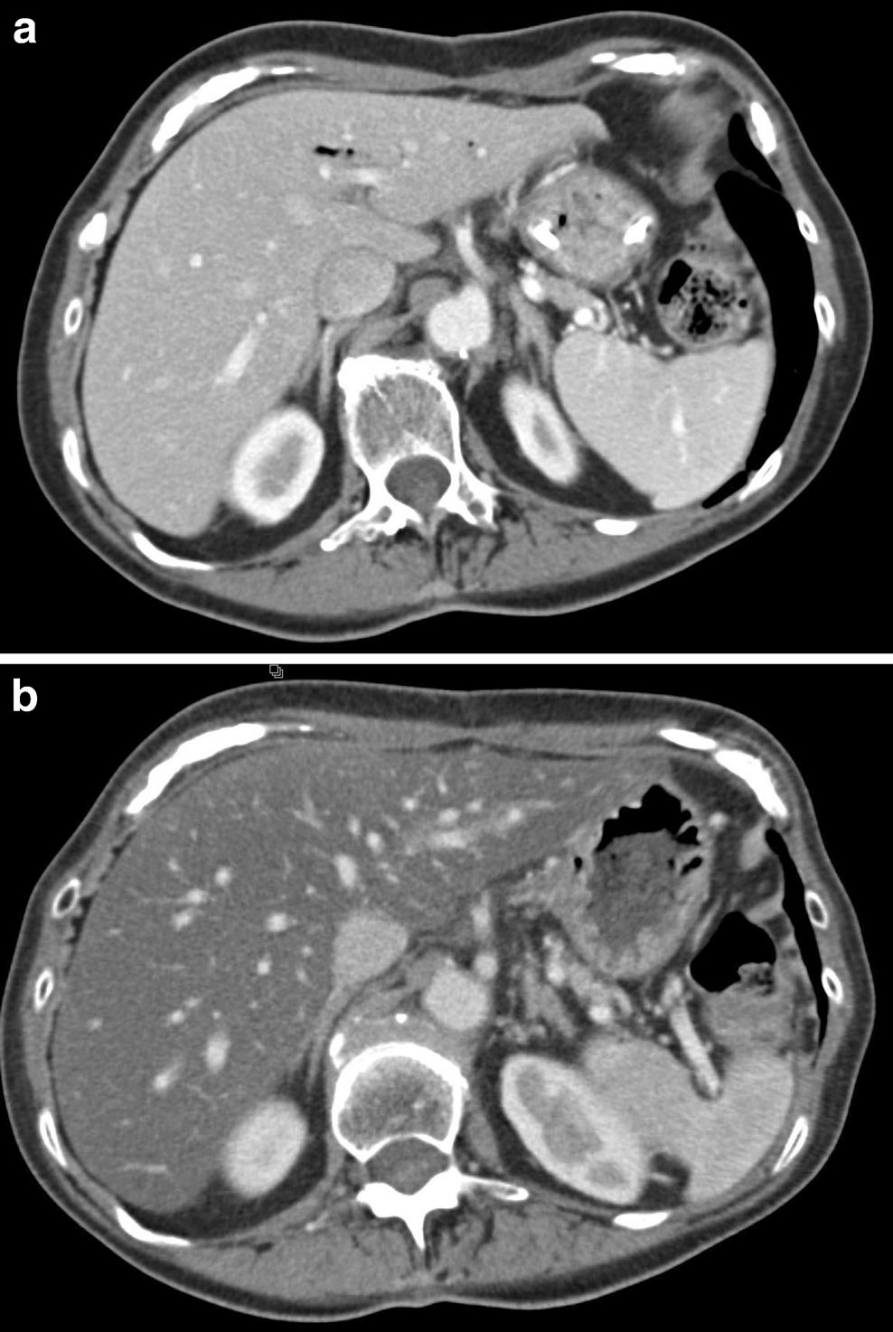
A 61-year-old male with colon cancer undergoing systemic treatment with oxaliplatin. **a** Axial contrast-enhanced CT pre-chemotherapy shows normal findings. **b** Axial CT status post 3 months of treatment with oxaliplatin shows development of diffuse fatty infiltration with hyperattenuating intrahepatic vessels consistent with steatosis

**Fig. 2:**
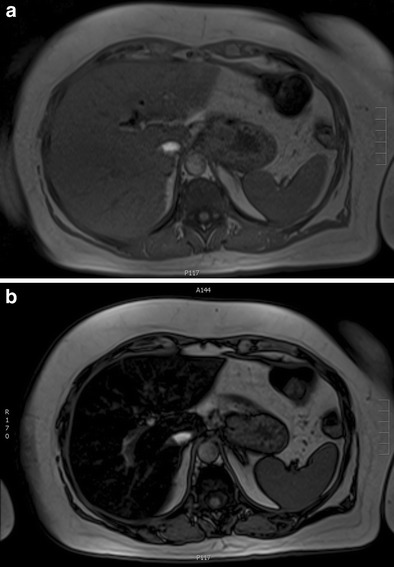
A 60-year-old female with colorectal cancer currently being treated with irinotecan. **a**, **b** Axial T1-weighted MR images in and out of phase, respectively, show significant signal drop in the liver consistent with steatosis

Chemotherapy-associated steatohepatitis (CASH) may be diffuse or focal and can be seen on ultrasound (US) as increased parenchymal echogenicity and beam attenuation [3]. Steatosis may also be visualised on computed tomography (CT) and is characterised by decreased parenchymal attenuation (Fig. 1) [4]. On magnetic resonance (MR) imaging, fatty liver can be detected by a drop in signal intensity on opposed-phase images compared with in-phase images. This is particularly useful in the diagnosis of focal hepatic steatosis (Fig. 2) [5, 6]. Detecting and reporting fatty changes on imaging in the chemotherapy patient is meaningful as it may prompt changes in treatment, especially in metastatic colorectal cancer patients who are planned for hepatic metastasectomy as underlying steatosis may increase the risk for post-operative complications [7]. Steatotic changes may be reversible with cessation of therapy and severe cases of CASH may require adjustment of surgical plans as there is increased hepatopathy, liver failure (5.8 % vs. 0.8 %), and mortality rate (14.7 % vs. 1.6 %) in these patients following partial hepatectomy [8, 9].

#### Sinusoidal obstruction syndrome

Hepatic sinusoidal obstruction syndrome (SOS) is an injury to the hepatic venous endothelium causing deposition of fibrous material within the venule walls and liver sinusoids; this deposition leads to obstruction of small intrahepatic vessels [10]. Clinical presentation of SOS includes hepatosplenomegaly, jaundice, abdominal pain, and ascites and can be characterised histologically by sinusoidal fibrosis, sinusoid dilation and congestion, and necrosis of pericentral hepatocytes [11, 12]. SOS is associated with systemic cancer therapies such as oxaliplatin, fluorouracil (5-FU), mercaptopurine (6-MP), and dacarbazine and may present as early as 1 to 3 weeks after initiation of therapy [13–15].

US findings of chemotherapy-induced SOS include ascites, gallbladder wall thickening, and hepatosplenomegaly as well as decreased flow through the portal vein on Doppler US [7]. CT reveals similar findings of hepatosplenomegaly, ascites, perioesophageal varices, and recanalisation of the umbilical vein [4]. Post-contrast enhancement CT and MR imaging commonly reveal patchy liver enhancement and narrowing of the main hepatic veins [16]. With detection of ascites, it is important to confirm the diagnosis of SOS as opposed to malignant ascites associated with peritoneal spread or metastasis [17]. Malignant ascites account for approximately 10 % of ascites cases; thus, the distinction between the two is crucial so that the appropriate treatment steps can be taken [17].

#### Pseudocirrhosis

Pseudocirrhosis describes changes in hepatic contour, such as the development of diffuse hepatic nodularity, in patients following chemotherapy treatment for hepatic metastases [18]. Pseudocirrhosis is most commonly found following chemotherapy with gemcitabine in patients treated for metastatic breast cancer [19]. While the majority of the clinical features associated with cirrhosis are absent in pseudocirrhosis, portal hypertension may be present [4]. Qayyum reports hepatic contour changes in 75 % of patients being treated for breast cancer metastasis, with 9 % of patients showing signs and symptoms of portal hypertension [20].

**Fig. 3:**
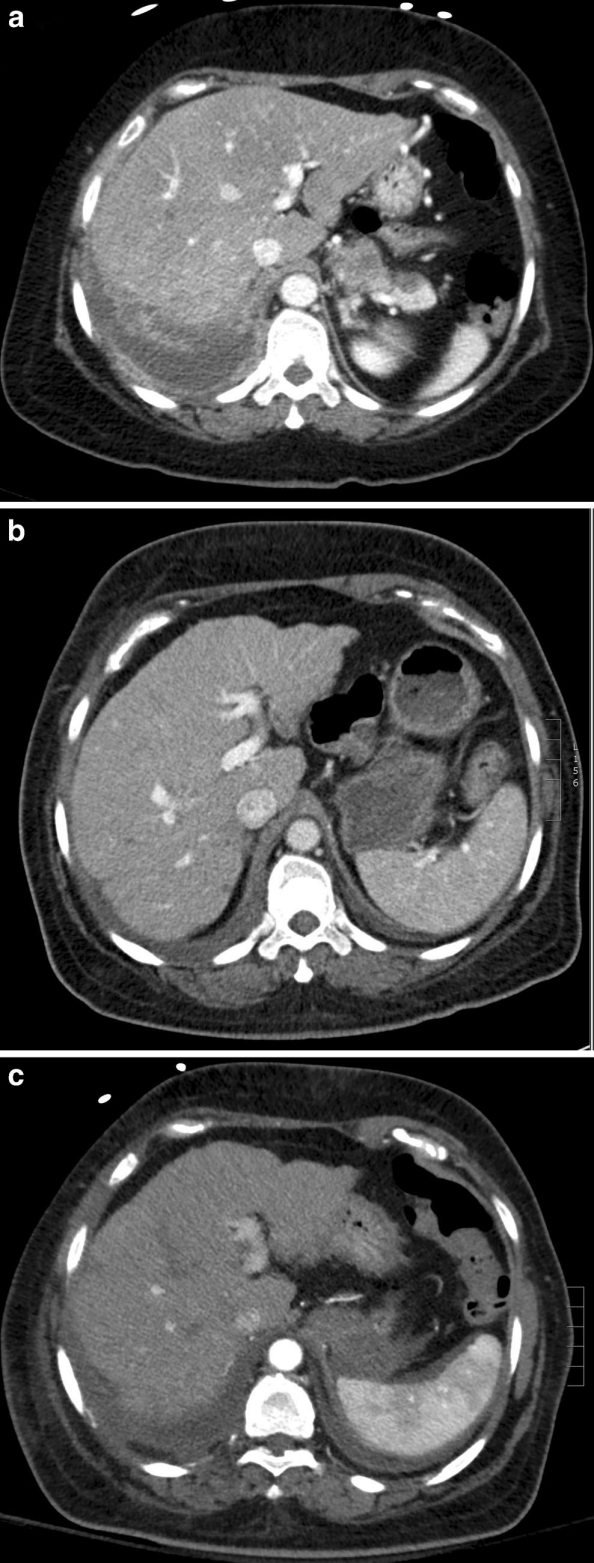
A 48-year-old female with metastatic breast cancer status post treatment with docetaxel and epirubicin. **a** Axial contrast-enhanced CT before chemotherapy shows normal-appearing liver with a smooth surface. **b** Repeat CT 6 months after initiation of treatment shows nodularity of the left hepatic lobe. **c** Repeat CT 7 months after initiation of treatment shows diffuse nodularity and capsular retraction consistent with pseudocirrhosis

The morphologic changes characteristic of pseudocirrhosis are initially detected between 4 and 8 months after therapy, may be diffuse or focal, and mimic liver cirrhosis, causing segmental volume loss, capsular retraction, fibrosis, and enlargement of the caudate lobe on US, CT, and MR imaging (Fig. 3) [18, 19, 21]. Additionally, the imaging findings of portal hypertension such as ascites, portosystemic venous collaterals, and splenomegaly may also be present [22]. CT is generally the modality of choice for detecting the features of pseudocirrhosis; however, MRI is more sensitive than CT for the follow-up of liver metastases and is recommended in these patients. While the exact mechanism underlying pseudocirrhosis is not fully understood, it has been linked with complications of hepatic encephalopathy, variceal bleeding, and portal hypertension [4]. Discontinuation of chemotherapy may be warranted if pseudocirrhotic complications cannot be managed conservatively [19].

#### Acute hepatitis

Recent literature has demonstrated an association between acute hepatitis and several targeted therapies such as anastrozole and lapatinib [8]. Additionally, reactivation of hepatitis B has been observed following treatment with rituximab, alemtuzumab, and infliximab [23]. Reactivation of hepatitis C has been observed with rituximab, alemtuzumab, and gemcitabine [24]. The presentation of acute hepatitis may range from asymptomatic to severe with nausea, poor appetite, vomiting, right upper quadrant pain, and jaundice with elevated AST and ALT. In the majority of patients, hepatitis is improved and reversible with temporary cessation of cancer therapy [2].

**Fig. 4:**
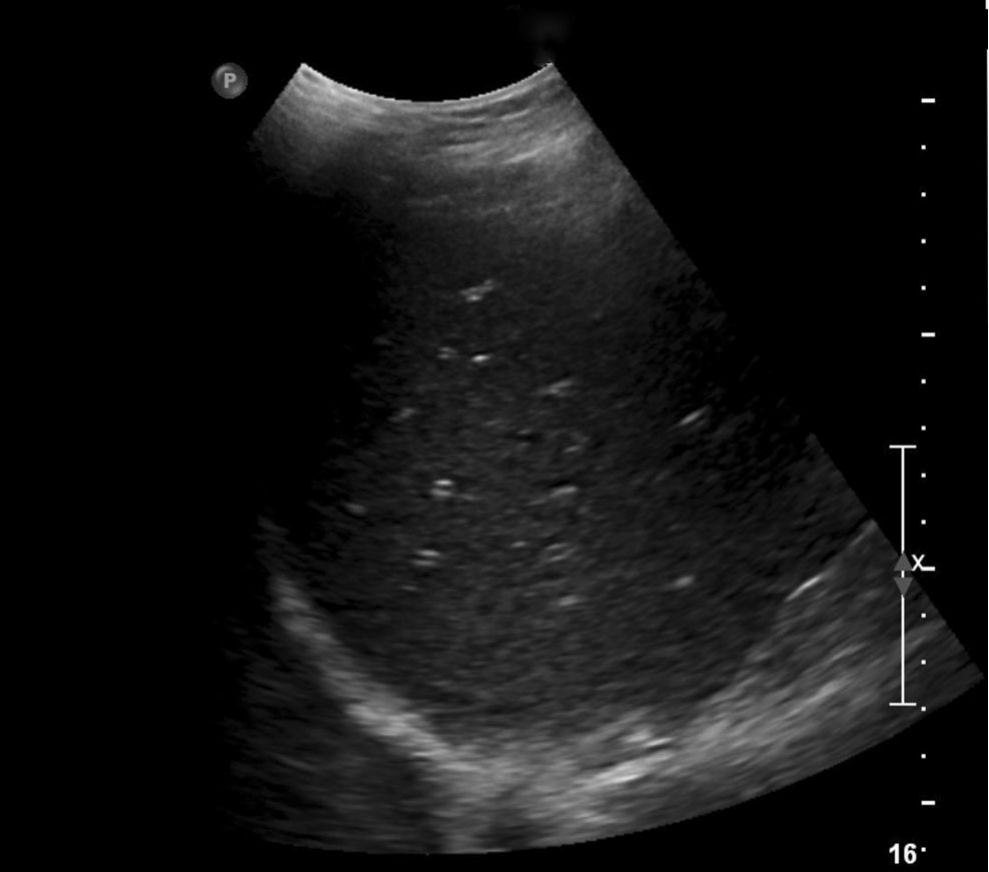
A 63-year-old female with metastatic breast cancer undergoing treatment with a drug regimen that includes lapatinib. Longitudinal US of the liver reveals diffuse starry sky appearance consistent with diffuse hepatic oedema and acute hepatitis

**Fig. 5:**
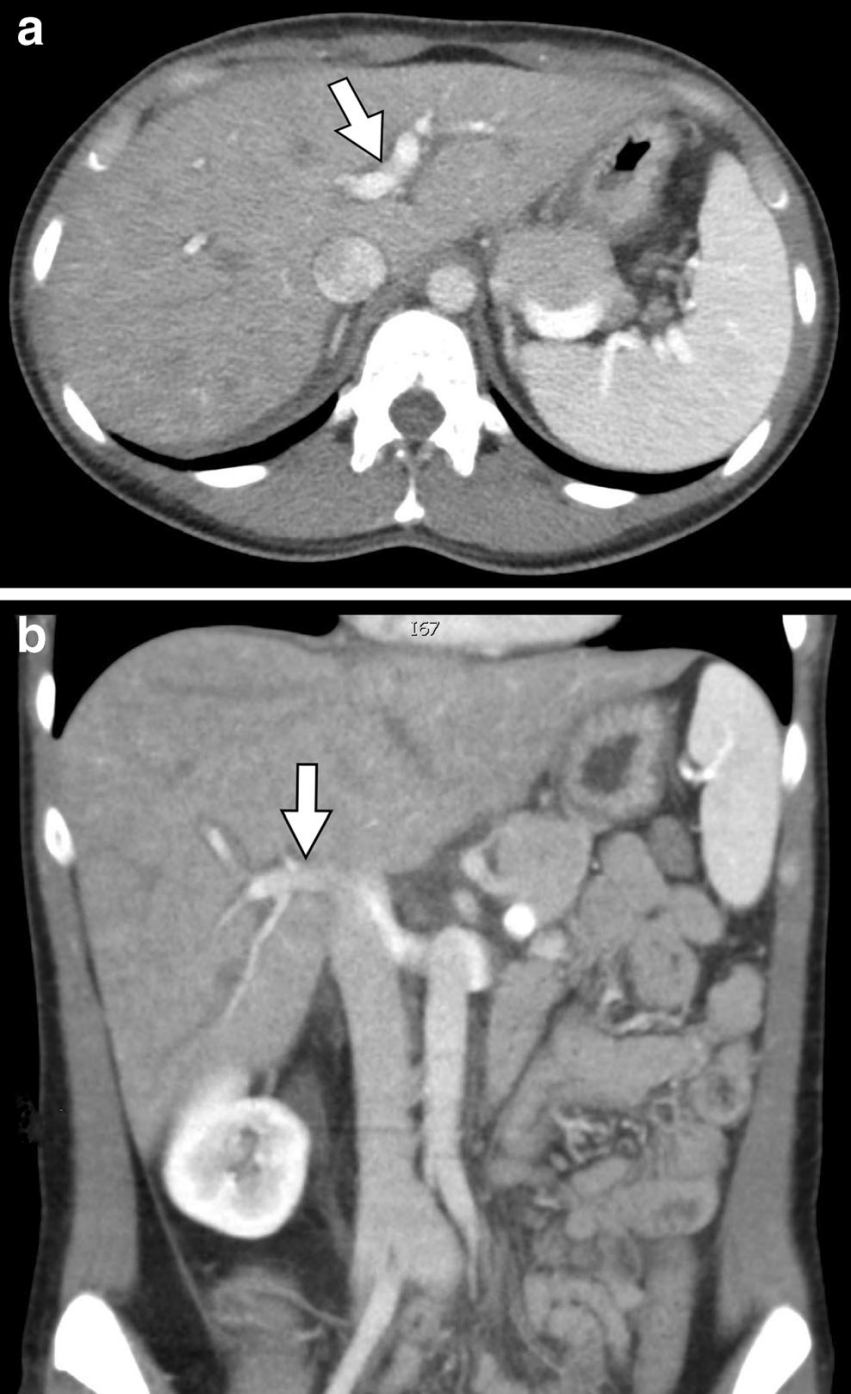
A 54-year-old male with acute myelogenous leukaemia treated with alemtuzumab. **a**, **b** Axial and coronal contrast-enhanced CT images, respectively, demonstrating hepatomegaly and periportal oedema (*arrows*) indicative of acute hepatitis

In patients with acute hepatitis, ultrasound demonstrates multiple echogenic foci within a relatively hypoechoic liver parenchyma, the so-called “starry sky” sign (Fig. 4) [25]. CT findings of acute hepatitis are nonspecific and include hepatosplenomegaly, thickened gallbladder wall, periportal oedema, and decreased liver enhancement (Fig. 5) [26].

### Pancreas

#### Pancreatitis

Chemotherapy-induced pancreatitis is well documented in the literature and is associated with several chemotherapeutic agents. These chemotherapeutic agents include L-asparaginase, carboplatin cisplatin, cytarabine, ifosfamide, paclitaxel, tretinoin, and vinorelbine [27, 28]. The onset of pancreatitis is variable and may range from hours to 1 month after drug administration [27].

Among the listed agents, chemotherapy-induced pancreatitis is most closely associated with asparaginase therapy with a prevalence of 2–16 % [7, 29]. L-asparaginase is a cytotoxic chemotherapeutic agent commonly used in the treatment of acute lymphocytic leukaemia. Additionally, a correlation between the targeted tyrosine kinase inhibitors sorafenib and sunitinib and pancreatitis has been described in recent literature [29]. Motzer et al. examined the effects of sunitinib in patients with metastatic renal cell carcinoma and reported elevated serum lipase in 30 % of patients without any other clinical signs of pancreatitis [30]. In another study, clinical pancreatitis was found in 3 of 451 patients on sorafenib with several other documented case reports of sorafenib-induced pancreatitis that resolved with discontinuation or reduction in sorafenib dosing [31–33].

**Fig. 6:**
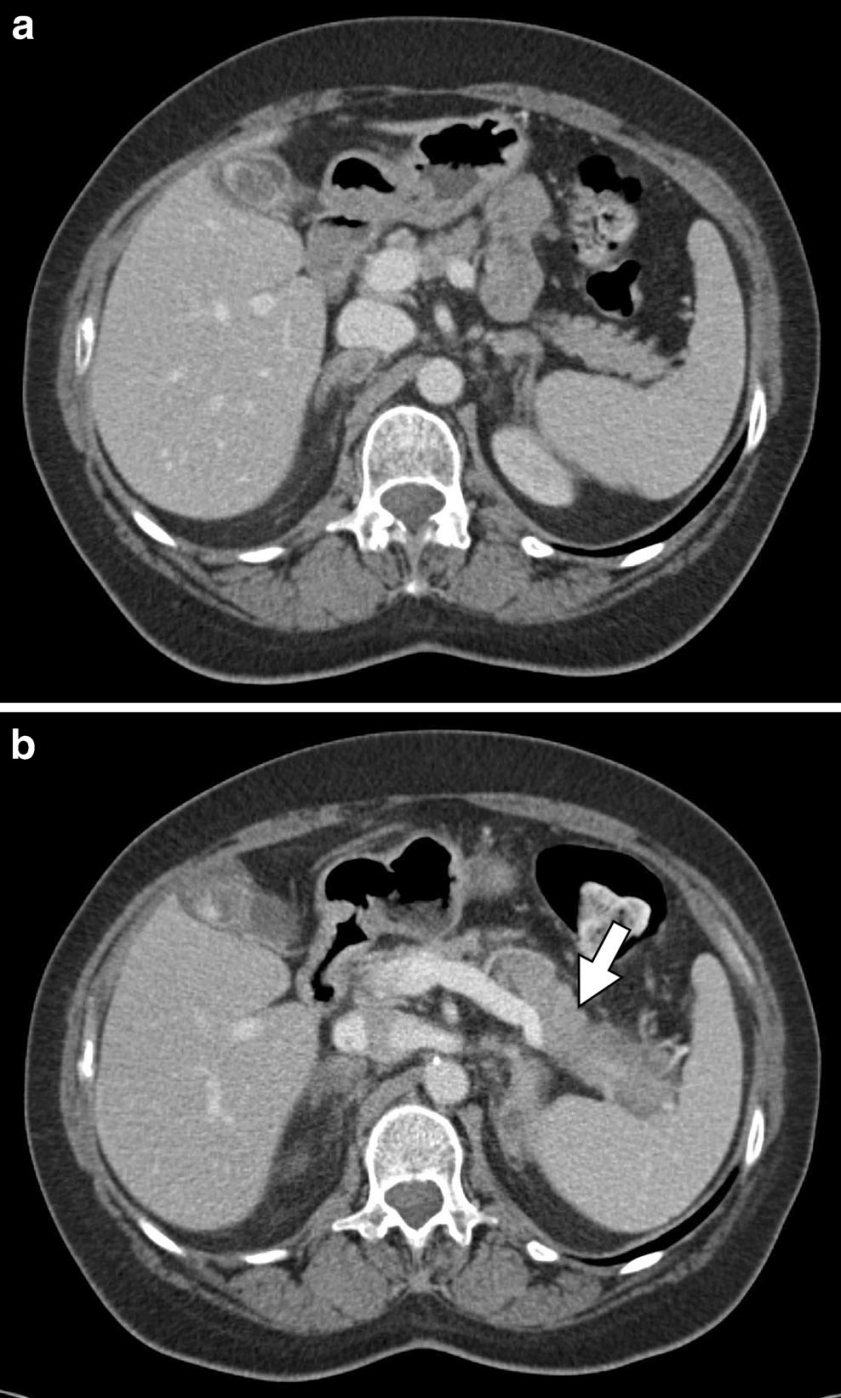
A 59-year-old female patient with stage IV non-small cell lung carcinoma (NSCLC) and known metastatic disease to the adrenal glands treated with carboplatin, premetrexed, and bevacizumab. **a** Axial contrast-enhanced CT shows a normal pancreas prior to chemotherapy. **b** Post-treatment axial contrast-enhanced CT demonstrating diffuse oedema and parenchymal enlargement (*arrow*) consistent with the development of acute interstitial pancreatitis

**Fig. 7:**
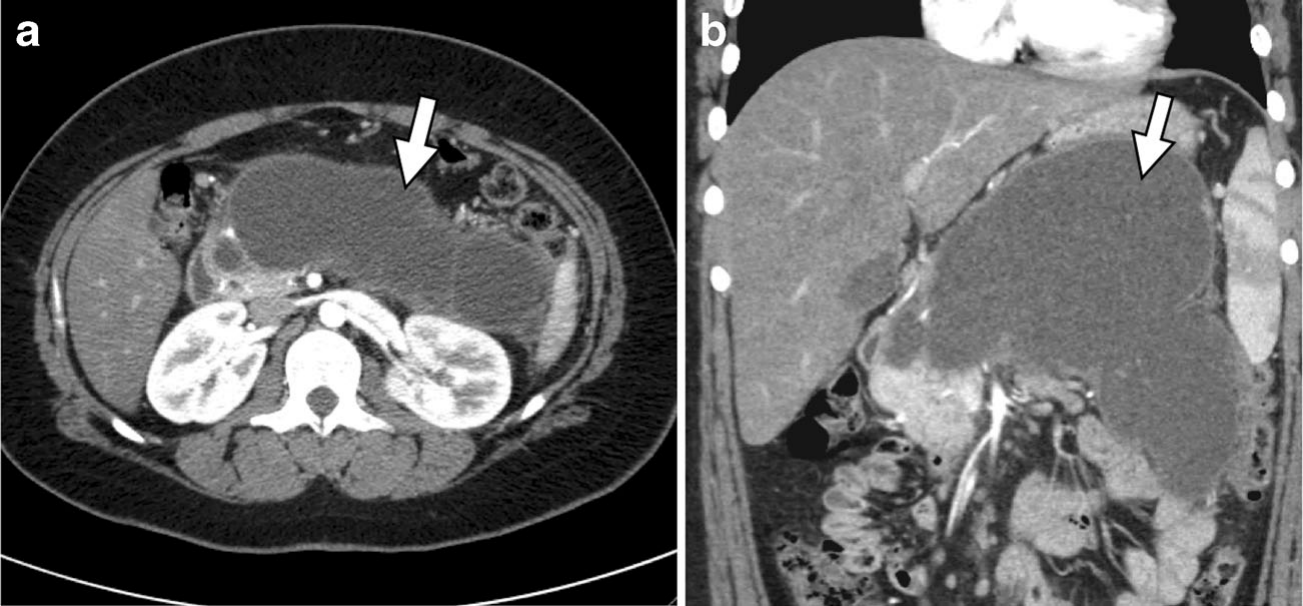
An 11-year-old male with acute lymphoblastic leukaemia on combination therapy that includes L-asparaginase. **a**, **b** Axial and coronal CT images, respectively, show “walled-off” necrosis (*arrows*) of the pancreas

For detecting and grading the severity of acute pancreatitis, multi-detector computed tomography (MDCT) is the modality of choice. Chemotherapeutic agents first cause acute interstitial pancreatitis, which may further progress to acute necrotising pancreatitis. On CT, acute interstitial pancreatitis may be characterised by fluid collections, peripancreatic fat stranding, focal areas of decreased attenuation, or diffuse oedema within the pancreas (Fig. 6) [34]. Similarly, necrotising pancreatitis is best seen on CT and has features of pancreatic inflammation and fluid collection in addition to the appearance of necrotic tissue (Fig. 7) [35]. Areas of decreased enhancement on CT and MR imaging can indicate necrosis of pancreatic tissue [36]. MRI can be helpful in grading the severity of acute pancreatitis, determining the presence and extent of necrosis, and may be more sensitive in the characterisation of peripancreatic collections [37, 38]. Visualisation of the pancreas via US imaging is often difficult because of patient body habitus and overlying bowel gas [39]. Thus, US evaluation of pancreatitis is often limited to the identification of peripancreatic fluid collection as well as hypoechoic lesions indicating necrotic changes [39].

#### Pancreatic atrophy

Pancreatic atrophy is a possible adverse effect of cancer therapy. In the literature, it has been reported that long-term use of sorafenib is correlated with pancreatic atrophy [40]. Ganten et al. explored the relationship between extended sorafenib therapy and pancreatic atrophy and reported a mean pancreatic volume loss of 25 % in hepatocellular carcinoma patients being treated with long-term sorafenib [40]. Hescott examined the pancreatic volume of two patients undergoing long-term sorafenib treatment and reported similar findings of 20 and 35 % irreversible reduction in pancreatic volume [41]. Atrophy is detectable as soon as 3 months after initiation of sorafenib therapy but may not present until as late as 2–3 years following treatment [40, 41].

**Fig. 8:**
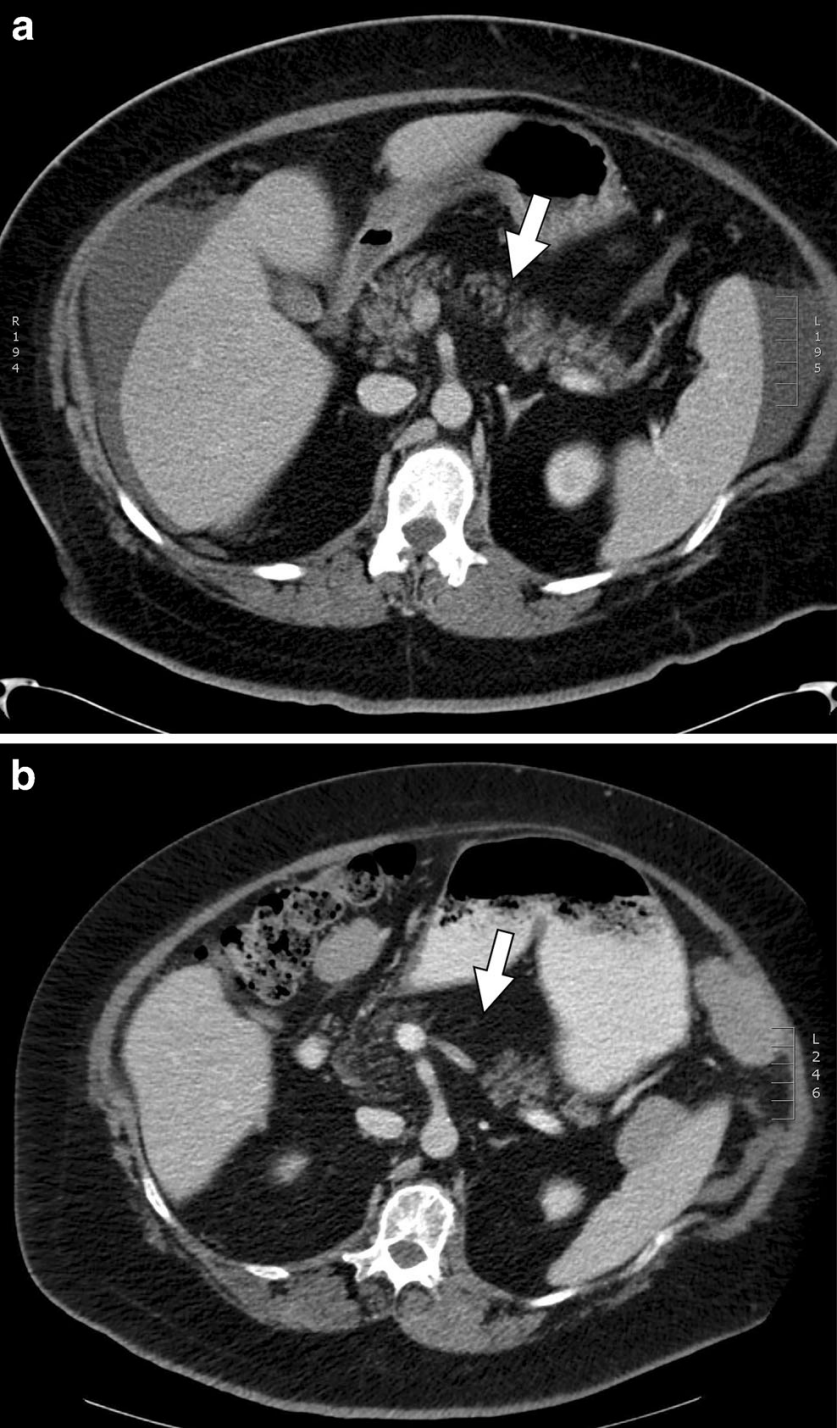
A 51-year-old female with stage IIIc ovarian cancer treated with bevacizumab. **a** Axial contrast-enhanced CT of the pancreas pre-chemotherapy with normal findings (*arrow*). **b** Repeat axial contrast-enhanced CT 11 months after initiation of chemotherapy shows marked pancreatic atrophy as well as fatty replacement (*arrow*)

Pancreatic atrophy can be best detected using CT and is often accompanied by fatty infiltration of the pancreas [42]. Measurement of pancreatic volume can be used to quantitatively monitor progression and/or improvement of atrophy and is commonly obtained by the “summation-of-areas” method in which the pancreatic tissue area from each CT slice is calculated and multiplied by slice thickness [43]. The volume of all the pancreatic slices is combined to yield the total pancreatic volume. Additionally, pancreatic size can also be evaluated by measuring the linear dimensions, such as the anteroposterior diameter, of the pancreatic head, body, and tail (Fig. 8) [44].

#### Other pancreatic changes

**Fig. 9:**
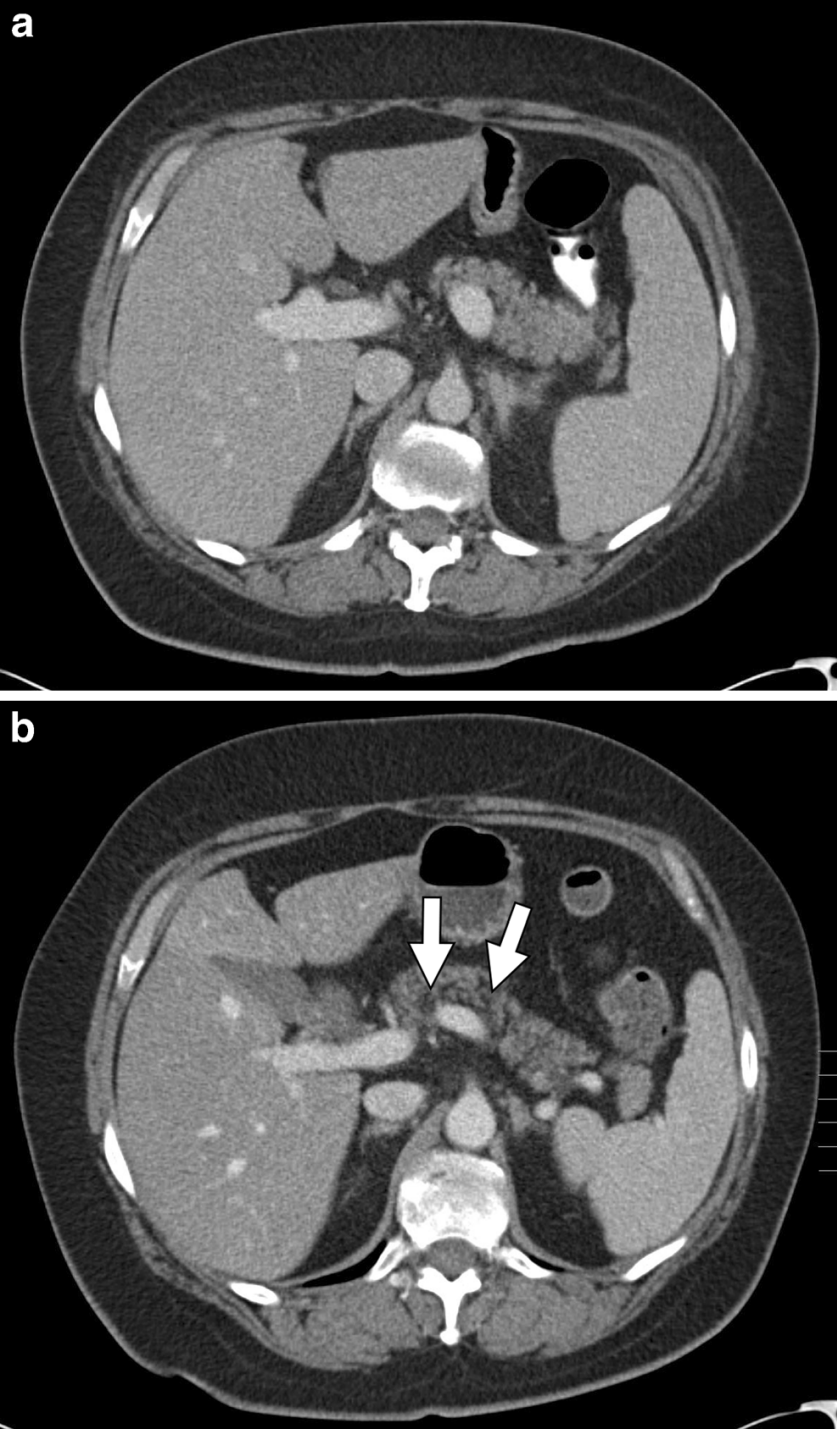
A 57-year-old female with leimyosarcoma treated with ifosfamide, mesna, and doxorubicin. **a** Pre-chemotherapy axial contrast-enhanced CT shows minimal fatty replacement of the pancreas. **b** Diffuse fatty replacement of the pancreas (*arrows*) is noted on axial contrast-enhanced CT performed 3 years later following cessation of chemotherapy

**Fig. 10:**
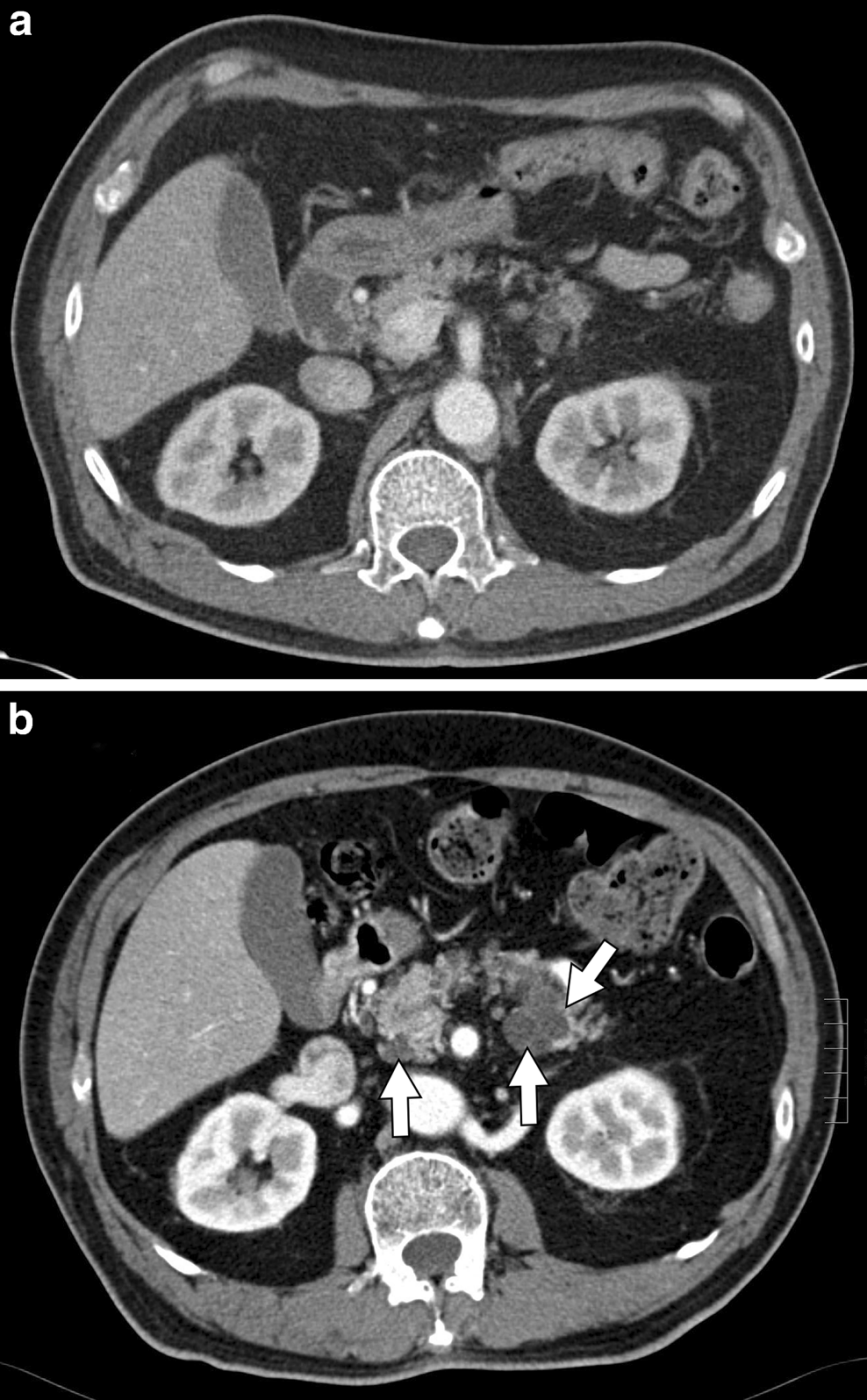
A 75-year-old male with lung adenocarcinoma receiving tarceva chemotherapy. **a** Axial contrast-enhanced CT image performed prior to initiation of chemotherapy treatment shows normal pancreatic features. **b** Axial contrast-enhanced CT performed following 2 years of chemotherapy shows the progression and development of multiple low-attenuating cystic lesions (*arrows*)

Other pancreatic changes that have been observed include fatty replacement of pancreatic tissue over the course of cancer therapy and cystic changes in the pancreas (Figs. 9 and 10). The evidence of the relationship of these findings to specific agents is lacking in the literature; however, these changes have been observed in our institution in patients following cancer therapy with no predisposing factors for the discussed findings.

### Biliary system

#### Acute acalculous cholecystitis

Cholecystitis is inflammation of the gallbladder. Symptoms include right upper quadrant abdominal pain, fever, nausea, and vomiting. There is growing evidence in the literature associating the use of targeted therapeutic agents with acute acalculous cholecystitis, particularly everolimus and sunitinib [45, 46]. Tirumani et al. examined the use of molecular-targeted therapies (sunitinib, bevacizumab, everolimus, and sorafenib) and associated gallbladder complications and found acute cholecystitis in 66 % of patients with variable onset between 2 weeks and 5 months [47]. These patients ultimately required dose reduction, temporary discontinuation of the therapy, or permanent discontinuation of the therapy with 50 % requiring either cholecystectomy or cholecystotomy [47].

**Fig. 11:**
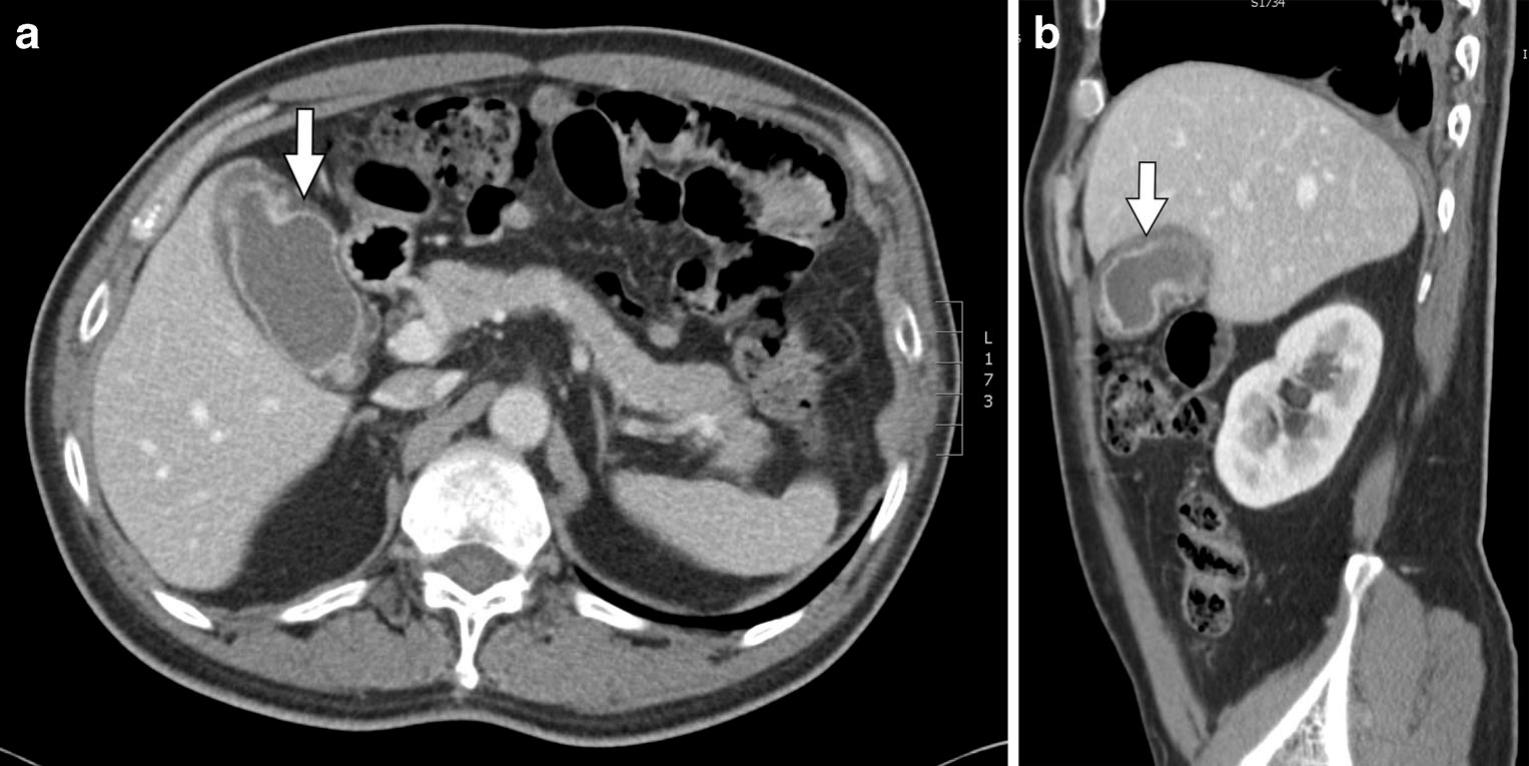
A 54-year-old male with stage IV non-small cell lung cancer status post chemotherapy treatment with cisplatin and premetrexed. **a**, **b** Axial and sagittal contrast-enhanced CT images, respectively, show a distended gallbladder with diffuse gallbladder wall thickening (*arrows*) consistent with acute acalculous cholecystitis

US is the imaging modality of choice to diagnose acute acalculous cholecystitis. Sonographic findings of therapy-induced acalculous cholecystitis include gallbladder wall thickening (>3 mm), gallbladder distension, and pericholecystic fluid in the absence of cholelithiasis [48]. Findings associated with cholecystitis on CT include gallbladder distension (>40 mm), fat stranding, hyperaemia, and pericholecystic free fluid (Fig. 11) [48].

#### Biliary inflammation

**Fig. 12:**
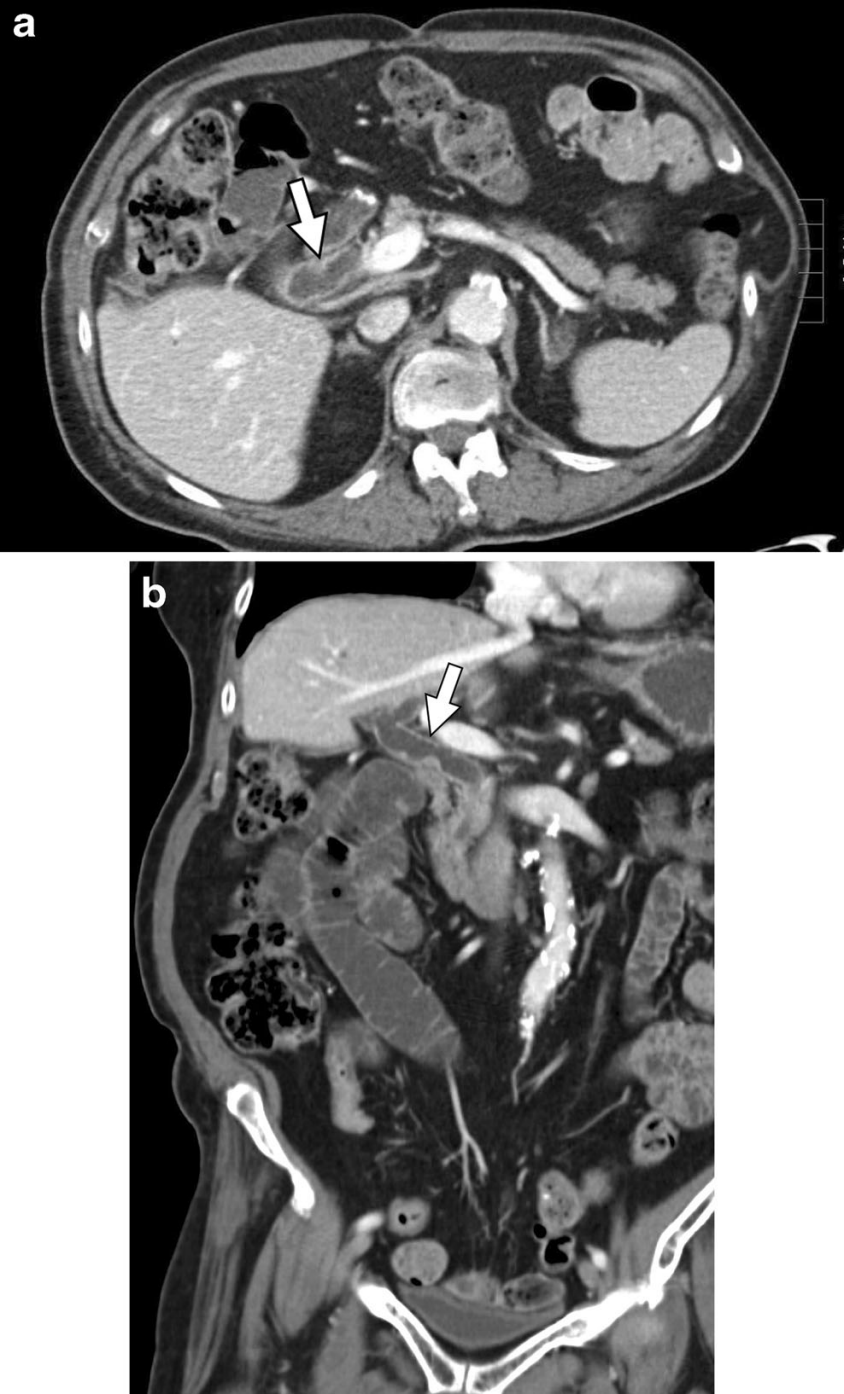
An 81-year-old female with stage IV recurrent squamous NSCLC treated with carboplatin, gemcitabine tarceva, and abraxane. **a**, **b** Axial and coronal contrast-enhanced CT images, respectively, demonstrate a mildly dilated common bile duct (*arrows*) with thickened enhancing walls consistent with biliary epithelial irritation

**Fig. 13:**
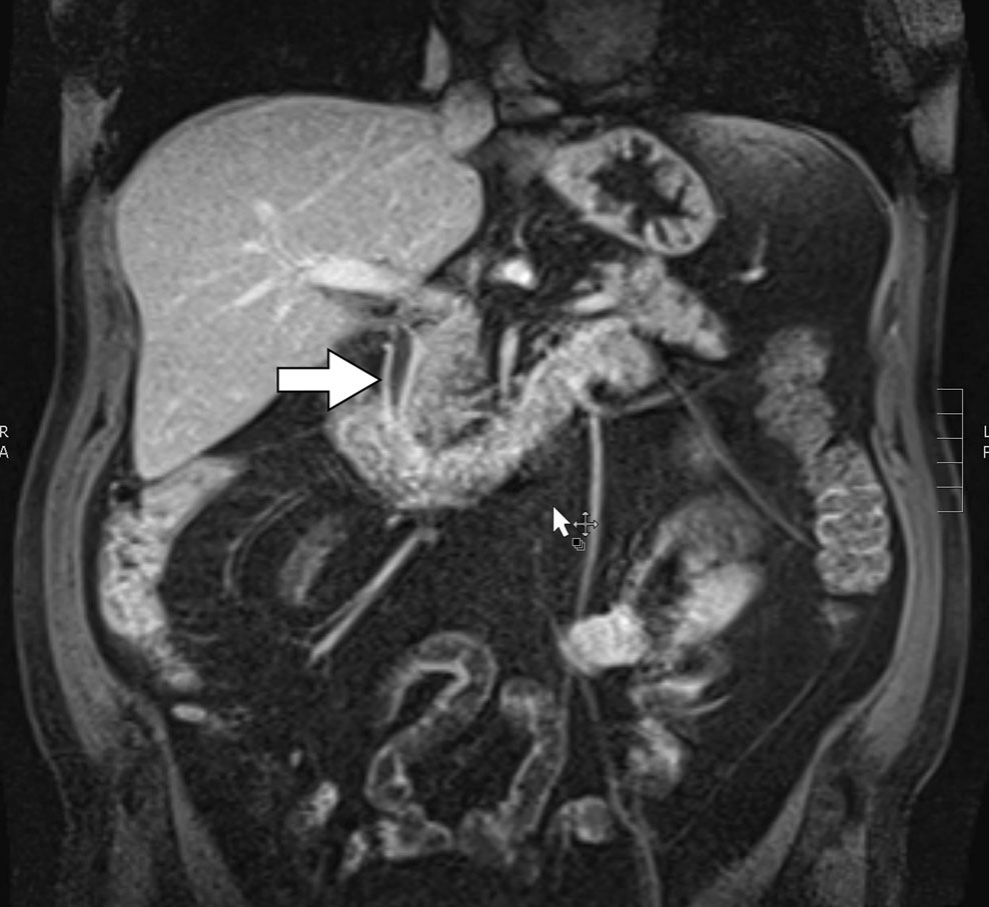
A 39-year-old male with non-Hodgkin’s lymphoma treated with doxorubicin. Coronal post-contrast MR image shows the thickened, enhancing biliary wall (*arrow*) of the common bile duct consistent with biliary inflammation

Oncologic agents are mainly excreted through the kidneys or the bile. Biliary excretion of certain oncologic agents may cause changes in the biliary epithelium leading to biliary enhancement on imaging. Common chemotherapeutic agents that are excreted through the bile include L-asparaginase, doxorubicin, epirubicin, and paclitaxel [49]. Common targeted therapeutics excreted through the bile include sorafenib and sunitinib [49]. Excretion of these therapeutic agents may have adverse effects on the epithelium causing irritation, thickening, and inflammation, which can be visualised as enhancement on CT and MR imaging (Figs. 12 and 13).

#### Biliary sclerosis

Chemotherapy-induced biliary sclerosis (CIBS) is a well-known toxicity associated with hepatic arterial infusion pump chemotherapy (HAIPC) with floxuridine [50]. Chemotherapeutic agents can cause CIBS through adverse toxicity on the biliary system or ischaemic changes to the pericholangitic venous plexus, leading to stricture of the biliary ducts [7]. Ito et al. examined the incidence of CIBS following HAIPC with floxuridine and found 5.5 % incidence in patients receiving HAIPC after hepatectomy and 2 % incidence in patients receiving HAIPC for unresectable disease [51]. CIBS can be managed with alternating infusion of an intrahepatic steroid or reduction in the chemotherapeutic dose while some cases may require stenting or dilatation [7].

The imaging findings of biliary sclerosis resemble those of primary sclerosing cholangitis and include a thickened and enhanced bile duct wall, bile duct stricture with the lumen measuring less than 3 mm, and periductal oedema [7, 52]. The gold standard imaging modality for detecting biliary sclerosis is endoscopic retrograde choloangiopancreatography (ERCP) [52]. ERCP is excellent for visualising the biliary tree and has high sensitivity for detecting dilatations and strictures of the biliary tree. Magnetic resonance cholangiopancreatography (MRCP) is non-invasive and offers similar imaging advantages as ERCP, but without the risks that ERCP has such as infection and bleeding [52]. The use of ERCP allows for interventional steps to be taken if necessary such as stenting and biliary stricture dilatation while MRCP reduces the risk of complications because of its non-invasive nature [52].

#### Biliary stasis

**Fig. 14:**
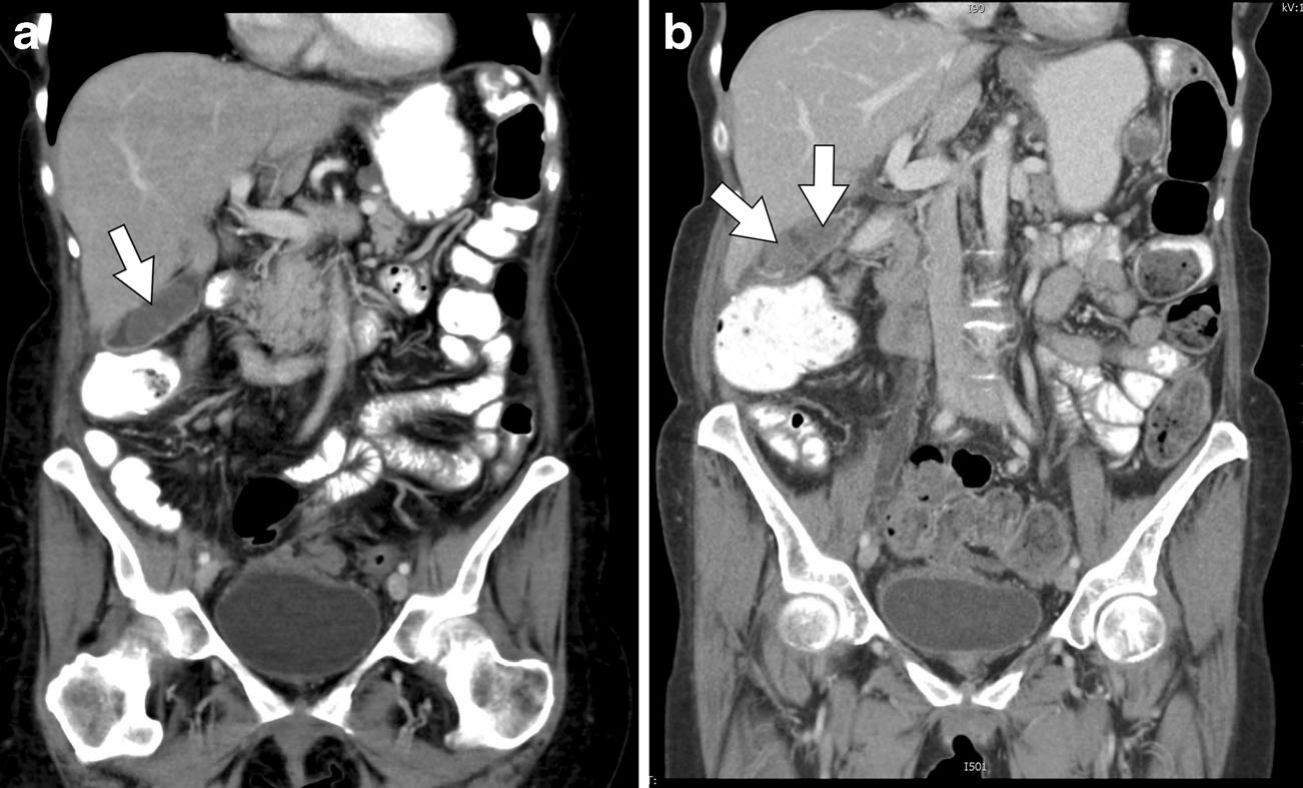
A 60-year-old female with recurrent ovarian cancer treated with a multi-drug chemotherapy regimen, which included gemcitabine. **a** Coronal contrast-enhanced CT following chemotherapy shows initial presentation of tumefactive sludge (*arrow*) within the gall bladder lumen. **b** Repeat coronal contrast-enhanced CT 6 months later shows progression of tumefactive sludge (*arrows*) and enhancing mildly thickened gallbladder wall changes, consistent with biliary stasis

In addition to the biliary enhancement, biliary stasis has been noted in our practice after initiation of tamoxifen and doxorubicin. Biliary stasis is a condition in which bile cannot be excreted from the liver into the duodenum and causes subsequent formation of biliary sludge and biliary dilatation [53]. Cholestasis may present with a number of symptoms including scleral icterus and pruritis; however, drug-induced cholestasis may be asymptomatic with elevated alkaline phosphatase as the only manifestation [54]. US is the primary modality used for diagnosing biliary stasis and is characterised by dilatation of the common bile duct (>7 mm) [53]. CT, cholangiography, and MR may be used to confirm biliary dilatation as well as the presence of tumefactive sludge (Fig. 14) [53]. Additionally, MR imaging is the modality of choice for non-invasive visualisation of the biliary tree and is useful in the diagnosis of many biliary conditions [53].

## Effects of localised therapy

Transcatheter arterial chemoembolisation (TACE) is a widely used treatment for hepatocellular carcinoma. TACE is a procedure that involves transarterial administration of a mixture of anticancer agents, such as doxorubicin or cisplatin, and iodised oil followed by embolising particles [55]. This allows for local administration of chemotherapeutic agents directly to tumours.

There are a number of imaging appearance changes that present following TACE. On CT, portions of the tumour that retain and accumulate iodised oil are necrotic and appear as non-enhancing foci while enhancing foci indicate viable tissue that may require additional treatment [55]. Kim et al. evaluated the value of the unenhanced phase in assessing HCC after TACE and found that the use of the unenhanced phase could improve the detection of viable tumour tissue with viable HCC appearing hyperattenuating or isoattenuating on the hepatic arterial phase and hypoattenuating on the unenhanced phase [56]. However, accumulation of iodised oil may generate considerable beam-hardening artefacts on CT that are not present on MRI [57]. In cases where there is a significant amount of iodised oil, MR imaging plays a vital role because of the artefact that appears on CT, resulting in difficulty detecting tumours using this modality [57]. On MRI, necrotic tissue will not demonstrate contrast enhancement while residual tumours will appear as hyperintense lesions [58].

Awareness of the normal imaging findings after TACE will allow for better recognition of pathologic changes associated with post-TACE complications. The adverse effects of TACE have been well documented in the literature and include hepatic abscess, hepatic failure, pancreatitis, and bile duct injury [59].

### Hepatic abscess

Hepatic abscess is a rare complication that can occur after TACE and may develop secondary to bacterial seeding of bilomas or from the necrotic core of an embolised hepatic tumour [60]. Marelli et al. found liver abscess formation in only 1.3 % of patients with VanderWalde reporting that patients with a history of bilioenteric anastomosis or biliary reconstruction are at greatest risk for developing abscesses [61, 62]. Woo et al. performed a study observing liver abscess formation after the TACE procedure with doxorubicin, iopamidol, and absorbable gelatin sponge embolisation in patients with bilioenteric anastomoses and found that abscesses formed in 12 of 25 patients, significantly higher than the 1.3 % reported for the general patient population [63]. Moreover, Woo et al. reported that particulate embolisation or oily portogram significantly elevated the incidence of liver abscess [63]. Hepatic abscess formation may appear at 2 weeks post TACE and most can be treated successfully with a combination of percutaneous drainage and antibiotic therapy [60, 62].

Hepatic abscesses can be diagnosed with the highest sensitivity on CT imaging and present as hypoattenuating lesions with peripheral rim enhancement; however, imaging of the post-TACE liver commonly reveals gas formation within the areas of embolisation, which may confound hepatic abscess diagnosis and require aspiration for accurate diagnosis [60, 64]. Additionally, MRI may play a helpful role in the detection, characterisation, and evaluation of liver abscesses because of its multiplanar capability and sensitivity to small differences in tissue composition [65].

### Hepatic failure

Hepatic failure following TACE is dependent on the baseline hepatic function, with a higher incidence of liver failure in patients with Child C liver disease compared with B and A [60]. Due to the increased risk of complications, most individuals with Child C liver disease will not be accepted for TACE. Huang et al. conducted a prospective study finding acute liver failure in 13.4 % of patients with HCC following TACE performed with doxorubicin, Lipiodol, and Gelfoam cube embolisation [66] Acute liver failure was previously established as increased bilirubin ≥2 mg/dl, increased Child-Pugh score ≥2, or ascites within 14 days of the procedure [66]. Additionally, Lu et al. investigated liver function damage following superselective TACE with low-dose versus conventional-dose anticancer drugs in HCC patients. Patients either received low or conventional dose mitomycin C, epirubicin, and carboplatin [66]. Anticancer drugs were administered with Lipiodol followed by either gelatin sponge or polyvinyl alcohol (PVA) particle embolisation [66]. Lu et al. found that both doses worsened liver function, which was evaluated with Child-Pugh scores, total bilirubin, albumin, and alanine aminotransferase [66]. However, the conventional dose caused more severe impairment of liver function, suggesting that hepatic failure from TACE may be dependent on chemotherapeutic agent dosing [66].

Acute hepatic failure is primarily diagnosed clinically and confirmed with laboratory testing. Prolonged PT/INR, elevated aminotransferases, elevated bilirubin, and decreased platelet count are among the abnormalities that may be detected with laboratory tests [67]. Although the role of imaging is often limited, signs suggestive of hepatic failure can be seen on US, CT, and MR imaging. On US, liver failure presents as increased hepatic echogenicity, ascites, nodularity, and segmental hypertrophy or atrophy [68]. Similarly, CT and MR can reveal ascites, surface and parenchymal nodularity, heterogeneous liver parenchyma, and segmental hypertrophy or atrophy [60, 68].

### Pancreatitis

Pancreatitis is an uncommon but severe complication of TACE. The incidence of this complication is rare, reported in only 1.7 % of TACE patients, and may occur because of reflux of chemoembolic agents to the pancreas [69, 70]. López-Benítez et al. investigated acute pancreatitis following TACE and found acute pancreatitis 24 h after the procedure in 15.2 % of patients with non-selected embolisation. The number of procedures and volume of embolic material were found to be the most significant factors associated with incidence [71]. TACE-induced pancreatitis can be treated in the same manner as pancreatitis from other causes [70].

Awareness of this complication following TACE warrants routine monitoring of serum amylase and lipase and complaints of abdominal pain may raise suspicions of acute pancreatitis related to TACE. As mentioned previously, pancreatitis may be characterised on CT by fluid collections, peripancreatic fat stranding, or diffuse oedema within the pancreas. Areas of low attenuation within the pancreas can indicate necrosis of pancreatic tissue.

### Bile duct injury

Bile duct necrosis, biliary stricture, and biloma development can occur following TACE because of ischaemic injury to the biliary plexus [60]. Sakamoto et al. examined 972 patients to investigate the incidence of biloma formation after TACE [72]; 3.6 % patients developed intrahepatic biloma following TACE therapy with the main risk factors being injection of a suspension of anticancer drugs versus a mixture, bile duct dilatation, and repeated chemoembolisations with a frequency of less than 3 months [72]. Miyayama et al. found main bile duct stricture with subsequent development of bile duct dilatation in 4 % of HCC patients after TACE performed with Lipiodol, epirubicin, and mitomycin C followed by gelatin sponge particles [73]. The main risk factor identified by Miyayama for development of main bile duct stricture following TACE is selective TACE of the caudate arterial branch and/or the medial segmental artery of the liver [73].

Biliary strictures and bilomas commonly require endoscopic or percutaneous drainage [60]. CT and MR can be used to diagnose bile duct complications and portal vein obliteration following TACE [74]. Bile duct injury can be seen as bile duct dilatation and areas of decreased attenuation, which is indicative of extrabiliary collection of bile [75]. MRCP allows for excellent evaluation of the biliary tree and fluid collections. When combined with a biliary contrast agent, MRCP is also able to detect and localise bile duct leaks [76].

## Conclusion

Cancer therapy includes a wide array of different types of treatment, which includes chemotherapy, targeted therapy, and catheter-directed chemoembolisation. The intrinsic toxicity of these therapies contributes to their efficacy in combating tumour growth, but also to the adverse effects they have on healthy tissues. These toxic agents commonly affect the liver, pancreas, and biliary system. The different treatment modalities and anticancer agents can cause sinusoidal obstruction syndrome, fatty liver, pseudocirrhosis, pancreatitis, pancreatic atrophy, cholecystitis, biliary sclerosis, and biliary inflammation. These changes may result in severe complications for patients and even death. It is important to note that the imaging findings associated with the cancer therapy complications discussed are often similar to the findings seen with other causes of complications unrelated to cancer therapy, making clinical information crucial when formulating treatment plans. Awareness and familiarisation with the imaging features associated with these cancer therapy-induced changes will allow for early detection of these complications and improved patient management and outcomes.
